# Supplementary material for: Female Sex Determination Factors in Ceratitis capitata: Molecular and Structural Basis of TRA and TRA2 Recognition
Source: Insects. 2023 Jul 4;14(7):605. doi: 10.3390/insects14070605 (PMC10380613; doi:10.3390/insects14070605)
Supplement: Supplementary file 1 [file insects-14-00605-s001.zip › insects-2370088-supplementary.pdf]

Supplementary material

Cc-TRA

1 50
MNMNITKASATTRKIRIEQNVPSGSVRKGPYAIERSVNPSEVVIKRRFGEGSKPLFQRDDIVVNPDNVVS
100
NVGAHFETQPKDRSNNSKEEVENQWRKERHKSTDSSSPERFRKHHSSNKSEHSNSGNNITTRHTKTHHPS
150 200
QENLNTASKRRDSSPPTNRRHRTPEKVPYFIDEIRERDRIRRKYGKRSTKSPSPPVMSSKFRRRRSYSRS
250
ISRSRSHSPARSKNRTHVYGSLSRRSSSVDRYIGGGRKRRRENLRTERDRDGQYRHHGHRSEEQERSRRG
300 350
RSPRARTRSRTRSRERSKHVRARNDERNKNLHGNHDELTNAELNQRNLTQPQIITIPVPVPADFLNYAYS
400
TWPTQTQWSHPMTPPPRYGAPAYHMPTILPATVMPPMRPALPPYGLPPQPMRYGGRGLRFPQQHGPRPWR
429
PNFRPKTHK

CcTRA-2

1 50
MSPRSRSRSISARRSYTKSPARRSNGRRRHSREKVYKSRSRSISRHPPSPPPPPTGRGGVRCSDASQSSS
100
TSLSPRQGRRMSRSRSRSPYDKRRGNREKPVQNRCIGVFGLSVYTTQQKIRDIFSRFGPIERIQVVIDAQ
150 200
TGRSRGFCFIYYDDIADAKAAKDACSGMEIDDRRIRVDYSTTQRPHTPTPGVYMGRHTRREREYNDRYRD
250
DYRPRRRSGSPFKNRNNYRNDRRRRYDRSRSRSYSPRRARY

Figure S1.
Sequences of CcTRA, and CcTRA2. Arg and Ser residues are highlighted. The folded RRM domain of CcTRA2 and the putative CcTRA2-binding domain of CcTRA are underlined.

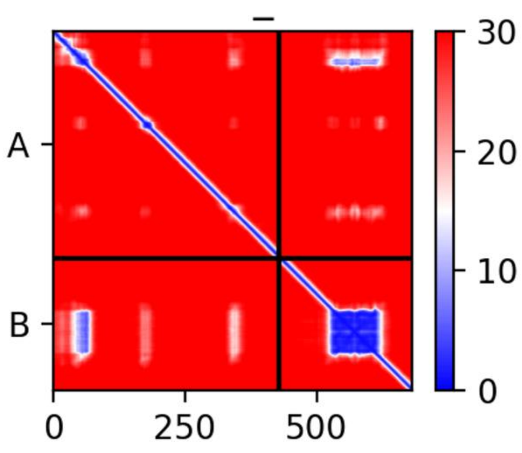

A

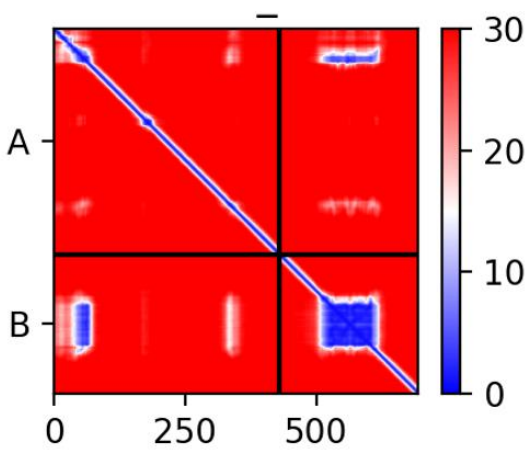

B

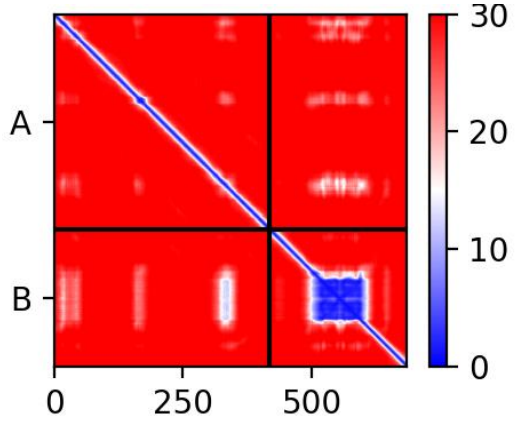

C

Figure S2.
Predicted Aligned Error matrices for the CcTRA/CcTRA2 (**A**) and CcTRA/DmTRA2 (**B**). On the vertical axis, the A and B letters identify the residues of CcTRa or CcTRA2/DmTRA2, respectively. The vertical bar reports the color code for the expected errors in Å . In the panel C, the PAE matrix obtained from the predition of a putative complex between CcTRA2 and a variant of CcTRA in which the region 54-64 was deleted is reported.

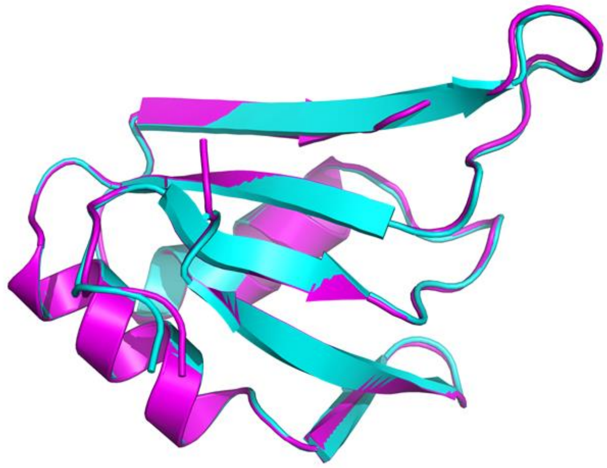

Figure S3.
Superimposition of the AF-predicted models of the RRM domain of CcTRA2 (cyan) with the DmTRA2 (magenta).
